# Early sexual initiation and multiple sexual partners among Vietnamese women: analysis from the Multiple Indicator Cluster Survey, 2011

**DOI:** 10.3402/gha.v9.29575

**Published:** 2016-02-29

**Authors:** Dinh Thai Son, Juhwan Oh, Jongho Heo, Nguyen Van Huy, Hoang Van Minh, Sugy Choi, Luu Ngoc Hoat

**Affiliations:** 1 Institute of Preventive Medicine and Public Health, Hanoi Medical University, Hanoi, Vietnam; 2 JW LEE Center for Global Medicine, Seoul National University College of Medicine, Seoul, South Korea; 3 Public Health Joint Doctoral Program, San Diego State University and University of California, San Diego, CA, USA

**Keywords:** early sexual initiation, multiple sexual partners, first intercourse, risky sexual behaviours, adolescent sexual behaviour

## Abstract

**Introduction:**

Under current HIV transmission mechanisms operating in Vietnam, women are seen as victims of their male partners. Having multiple sexual partners is one of the well-known risk factors for HIV infection. However, little is known about women's risky sexual behaviour and their vulnerability to HIV in Vietnam. This study aims to explore association between early sexual initiation and the number of lifetime sexual partners in Vietnamese women. Although the Vietnamese culture is socially conservative in this area, identifying women's risky sexual behaviour is important for the protection of women at risk of HIV and other sexually transmitted diseases.

**Design:**

A total of 8,791 women, who reported having had sexual intercourse, were included in this analysis of data from the 2011 Multiple Indicator Cluster Survey in Vietnam. Data were collected using two-stage strata sampling, first at the national level and second across six geographical regions (*n*=8,791). Multivariable logistic regressions describe association between early initiation of a sexual activity and lifetime multiple sexual partners.

**Results:**

Early sexual intercourse was significantly associated with having lifetime multiple sexual partners. Women who were aged 19 or younger at first sexual intercourse were over five times more likely to have multiple sexual partners, compared with women whose first sexual intercourse was after marriage; aged 10–14 years (OR=5.9; 95% CI=1.9–18.8) at first intercourse; and aged 15–19 years (OR=5.4; 95% CI=4.0–7.2) at first intercourse. There was significant association with having multiple sexual partners for women of lower household wealth and urban residence, but the association with educational attainment was not strong.

**Conclusions:**

The study results call for health and education policies to encourage the postponement of early sexual activity in young Vietnamese women as protection against risky sexual behaviour later in life.

## Introduction

Evaluating sexual and reproductive health (SRH) services and access to HIV prevention, treatment, care, and support are some of the key targets of the Millennium Development Goals (MDGs) (1). Understanding sexual behaviour among women may help to improve their sexual health and encourage them to avoid sexually transmitted diseases (STDs), unwanted pregnancies, and unsafe abortions (2). The most frequently examined indicator of risky sexual behaviour in the literature is early (young age) sexual intercourse (3). In many countries, women's age at first intercourse has decreased in recent years. For example in Britain, an increasing proportion of women are reporting that their first intercourse occurred before age 16 (4).

Early first sexual intercourse is a public health issue. Previous studies indicate that the earlier the woman's first sexual intercourse, the more likely it is that she will have unwanted pregnancies (4, 5) or acquire HIV or other STDs (6). Early sexual intercourse is commonly defined as having had first sexual intercourse before age 15 (7, 8). Young people do not always have sufficient negotiation skills and/or the power to ensure consistent and effective use of condoms (9). Studies show that, of the women who report having had early sexual intercourse, a high proportion also report having two or more sex partners in the previous 3 months; using alcohol/drugs during their last sexual intercourse; not using a condom at their last sexual intercourse; having been pregnant; having been forced to have sex; having been involved in physical intimate partner violence in the previous year and displaying antisocial behaviour in later life (10–13). Health risks such as HIV infection and STDs among women who have had multiple sexual partners are well known (10–12).

A few studies have been conducted on the age at first sexual intercourse and/or having multiple sexual partners in Vietnam. In one such study in Haiphong province, the mean age at first intercourse was 22.7 years (SD 22.4 years) and 9.2% of women had multiple partners (14). Another study in a mountainous north-eastern area of Vietnam (Quang Ninh Province) showed that the proportion of married women who reported having sexual intercourse before marriage was between 4 and 7% depending on the district (15).

Despite the growing body of research on first sexual intercourse, little is known about the association between early first sexual intercourse and later sexual behaviour, including having multiple sexual partners, or factors associated with early first sexual intercourse among Vietnamese women. The role played by young people in Vietnam in the spread of HIV and STDs from high-risk groups to the general population had been described in previous studies (14). Evidence of association between early first sexual initiation and later at-risk sexual behaviour can inform policy, education and interventions in SRH. The objective of this study of women in Vietnam is to describe the association between early sexual initiation and the number of lifetime sexual partners.

## Methods

Data were obtained from the 2011 Multiple Indicator Cluster Survey (MICS) in Vietnam. This cross-sectional survey was conducted by General Statistics Office of Vietnam at the national level for urban and rural areas and for Vietnam's six regions: Red River Delta, Northern Midlands and Mountain areas, North Central area and Central Coastal area, Central Highlands, South East, and the Mekong River Delta. Two-stage strata (urban and rural) sampling was used to select 12,000 household representatives of which 11,642 were present at the time of the survey. The first stage was carried out using the probability proportional to size sampling method. In the second stage, 20 households within the selected census enumeration areas were selected randomly from the list of all households. From the interviewed households, 11,663 out of 12,115 women (aged 15–49 years) completed the interviews. After excluding two women who had missing data for age at first sexual intercourse, a total of 8,791 women who reported having had sexual intercourse were selected for this analysis. Data were collected between 29 November 2010 and 26 January 2011.

The independent variable was ‘age at first sexual intercourse’. The question used to derive this variable was, ‘How old were you when you had sexual intercourse for the very first time?’ The variable was grouped into five categories: 1) aged between 10 and 14; 2) aged between 15 and 19; 3) aged between 20 and 24; 4) aged between 25 and 42; 5) the reference group, first intercourse with husband.

The binary dependent variable was ‘multiple’ (i.e. more than two) sexual partners over the lifetime (*n*=8,790). The question used to derive this variable was, ‘In total, with how many different people have you had sexual intercourse in your lifetime?’

Sociodemographic factors used to describe the study sample are: age group (15–19 years, 20–24 years, 25–29 years, 30–34 years, 35–39 years, 40–44 years and 45–49 years); highest educational level (preschool, primary, lower secondary, upper secondary, professional school, college/university); living area (urban, rural); region (Red River Delta; Northern Midlands and Mountain areas; North Central area and Central Coastal area; Central Highlands; South East and the Mekong River Delta); marital status (currently married, living with a man, never married or not in union); husband or partner had other wives (yes or no); number of husband's other wives (continuous variable); living with any sons or daughters (yes or no); ethnicity of the household head (Kinh or non-Kinh) and household wealth quintiles (poorest, second, middle, fourth, and richest quintile). To derive the household wealth quintiles, principal component analysis was performed by using assets associated with household wealth as follows: water sources, toilet facility, housing, fuel types for cooking, electricity, bank account, durable goods (such as radio, TV, refrigerator, fixed telephone, watch, mobile phone, bicycle, motorcycle, boat with motor, and car), animals (such as buffalo, cattle, horse, donkey, goat, sheep, chicken, and pig). The study sample was also described by age at first sexual intercourse, condom use during first sexual intercourse (yes, no, or do not remember), and the dependent variable, more than one sexual partner over the lifetime. Observations and percentages with 95% confidence intervals (CIs) are reported****.

Multivariable logistic regression was carried out to describe association between early sexual initiation and multiple sexual partners over the lifetime. Three models are shown. Model 1 is unadjusted with age at first sexual intercourse as the independent variable and the reference group, first sex with current partner. Model 2 adjusted for age (reference group: 15–19 years), ethnicity of household head (reference: non-Kinh), education (reference: primary or less), marital status (reference currently married), living with children (reference: living alone), the number of husband's other wives (continuous variable), and household wealth quintiles (reference: poorest). Model 3 additionally adjusted for living area and region. Statistical significance was set at *p*<0.05. Data analysis was performed using STATA software version 11 for windows.

## Results

A total of 8,791 women were included in the study sample. Characteristics of the participants are shown in Tables 1 and 2. Women aged 15–19, 20–24, 25–29, 30–34, 35–39, 40–44, and 45–49 constituted 2.0%, 9.6%, 17.6%, 18.8%, 18.1%, 18.3%, and 15.7% of the study population, respectively. Nearly half of the women completed lower secondary education (44.5%), and for 20.8% of women primary was their highest level of formal education; only 10.3% of women studied at college or university, and 6% of women attended professional schools. The number of women living in urban areas was 3,737 (42.5%), whereas there were 5,054 women living in rural areas (57.5%). Most of the participants were currently married (90.2%). About 84% of the sample had household heads of Kinh ethnicity. Among 204 women who reported on the number of previous wives that their husbands had had, 69.3% reported only one other wife.

**Table 1:** Socio–demographic characteristics of Vietnamese women MICS 4 – 2011 (*n*=8,791)

Variables	*n*	%	95% CI
Age group (in years)			
15–19	176	2	1.7–2.3
20–24	842	9.58	9.0–10.2
25–29	1,545	17.57	16.8–18.4
30–34	1,651	18.78	18.0–19.6
35–39	1,587	18.05	17.3–18.9
40–44	1,606	18.27	17.5–19.1
45–49	1,384	15.74	15.0–16.5
Highest education level			
Preschool	1	0.0	0.0–0.1
Primary	1,712	20.8	19.9–21.7
Lower secondary	3,668	44.5	43.4–45.6
Upper secondary	1,515	18.4	17.6–19.2
Professional school	497	6.0	5.5–6.6
College/university	851	10.3	9.7–11.0
Living area			
Urban	3,737	42.5	41.5–43.5
Rural	5,054	57.5	56.5–58.5
Region			
Red River Delta	1,310	14.9	14.2–15.7
Northern Midlands and Mountain areas	1,618	18.4	17.6–19.2
Northern Central area and Central Coastal area	1,362	15.5	14.8–16.3
Central Highlands	1,555	17.7	16.9–18.5
South East	1,466	16.7	15.9–17.5
Mekong River Delta	1,480	16.8	16.1–17.6
Marital status			
Currently married	7,932	90.2	89.6–90.8
Living with a man	260	3.0	2.6–3.3
Never married/not in union	599	6.8	6.3–7.4
Husband/partner had other wives			
Yes	204	2.5	2.2–2.9
No	7,985	97.5	97.1–97.8
Living with any sons or daughters			
Yes	7,732	94.6	94.0–95.0
No	445	5.4	5.0–6.0
Ethnicity of household head			
Kinh	7,372	83.9	83.1–84.6
Non-Kinh	1,419	16.1	15.4–16.9
Wealth quintiles			
Poorest	1,737	19.8	18.9–20.6
Second	1,468	16.7	15.9–17.5
Middle	1,649	18.8	18.0–19.6
Fourth	1,882	21.4	20.6–22.3
Richest	2,055	23.4	22.5–24.3
	Mean	SD	Range
Number of husband's other wives	0.03	0.21	0–3

**Table 2:** Characteristics of first sexual behaviour and number of lifetime sexual partners among Vietnamese women MICS 4 – 2011

	*n*	%	95% CI
Age of first sexual intercourse (*n*=8,791)			
First sex with the current partner	5,464	62.2	61.1–63.2
10–14	34	0.4	0.3–0.5
15–19	1,184	13.5	12.8–14.2
20–24	1,564	17.8	17.0–18.6
25–42	545	6.2	5.7–6.7
Condom use during first sexual intercourse (*n*=3,327)			
Yes	139	4.18	3.55–4.92
No	3,184	95.7	94.95–96.34
Do not remember	1	0.03	0.00–0.02
Women had more than one sexual partner over their lifetime (*n*=8,790)			
Yes	347	3.95	3.56–4.37
No	8,443	96.05	95.62–96.43

Among the women who reported sexual experience, those who had had their first sexual intercourse at 20–24 years of age were the largest single group (17.8%, 95% CI 17.0–18.6%) (Table 2). The proportion of women who reported having sexual intercourse for the first time before age 15 was only 0.4% (95% CI 0.3–0.5%). Only 4.2% of women reported using a condom during the first sexual intercourse (95% CI: 3.55–4.92). The proportion of women who had more than one sexual partner during their lifetime was 4.0% (95% CI: 3.55–4.92).

Table 3 shows the significant association between early sexual initiation and multiple sexual partners over the lifetime in three logistic regression models. Exposure to sexual intercourse early in life is an important factor that increases the likelihood of having multiple sexual partners. These associations are quite robust and statistically significant in all three models, although the strength of association attenuates in models 2 and 3. In model 1 (unadjusted), the odds ratio for age at first sexual intercourse from 10 to 14 years was 8.1 (95% CI: 3.1–21.3). However, this drops in models 2 and 3 to 6.2 (95% CI: 2.0–19.6) and 5.9 (95% CI: 1.9–18.8) respectively. Women who were richer, currently married, living with their children, had spouses who had no other wives, or were living in rural areas were less likely to have multiple sexual partners.

**Table 3:** Crude and adjusted logistic regression of association between age at first sexual intercourse and multiple sexual partners, Vietnamese women MICS 4 – 2011 (*n*=8,790)

	Model 1		Model 2		Model 3	
			
	OR	95% CI	OR	95% CI	OR	95% CI
Age of first sexual intercourse (ref: first sex with the current partner)						
10–14	8.1***	3.1–21.3	6.2**	2.0–19.6	5.9**	1.9–18.8
15–19	5.2***	4.0–6.8	5.4***	4.0–7.1	5.4***	4.0–7.2
20–24	2.8***	2.1–3.7	2.6***	1.9–3.5	2.6***	1.9–3.5
25–42	2.0**	1.2–3.1	1.2	0.8–2.1	1.2	0.7–1.9
Age (ref: 15–19)						
20–24			1.9	0.6–5.6	2.0	0.7–6.1
25–29			4.8	1.7–13.6	5.1**	1.8–14.7
30–34			7.2***	2.6–20.4	7.9***	2.8–22.6
35–39			7.0***	2.5–19.9	7.7***	2.7–22.2
40–44			8.3***	2.9–23.3	9.0***	3.1–25.7
45–49			7.1***	2.5–20.0	7.8***	2.7–22.5
Ethnicity of household head (ref: non-Kinh)						
Kinh			1.4	1.0–2.0	1.5	1.0–2.2
Education (ref: ≤primary)						
Lower secondary			0.6**	0.5–0.9	0.7*	0.5–0.9
≥Upper secondary			0.7	0.5–1.1	0.8	0.6–1.2
Marital status (ref: currently) married						
Formerly married			4.4***	3.3–6.1	4.1***	3.0–5.7
Never married			10.1***	5.7–17.8	10.0***	5.6–17.8
Wealth quintile (ref: richest)						
Fourth			0.9	0.6–1.4	1.0	0.7–1.5
Middle			1.0	0.7–1.5	1.4	0.9–2.1
Second			1.3	0.9–1.9	1.9**	1.2–2.9
Poorest			1.1	0.7–1.7	1.8*	1.1–3.0
Living with children (ref: living alone)						
Cohabiting			0.4***	0.3–0.6	0.4***	0.3–0.6
Number of other wives			5.4***	4.2–6.9	5.5***	4.3–7.2
Living area (ref: rural)						
Urban					1.5**	1.1–2.0
Region (ref: Red River Delta)						
Northern Midlands and Mountain area					1.0	0.6–1.7
Northern Central and Central Coastal area					1.0	0.6–1.6
Central Highlands					1.6*	1.0–2.6
South East					1.7*	1.1–2.7
Mekong River Delta					1.1	0.7–1.8
*R* ^2^	0.05		0.16		0.17	

* *p*<0.5

** *p*<0.01

*** *p*<0.001.

OR=odds ratio; CI=confident intervals.

Northern Midlands and Mountain=Northern Midlands and Mountain areas.

Northern Central and Central Coastal=North Central area and Central Coastal area.

## Discussion

This study shows a strong association between early sexual initiation and having multiple sexual partners over the lifetime among women in Vietnam. Considering evidence of the health risks of having multiple sexual partners, including the spread of HIV and other STDs, these findings shed light on the importance of strengthening health education not only for adolescent girls but also for boys.

In this study, the percentage of women whose first sexual intercourse was before age 15 was 0.4%. This result is higher than that of a similar study in Macedonia (0.1%) (5) but lower than reported in Canada (13%) (16). However, definitions of early first sexual intercourse vary widely making it very difficult to compare results across studies (8). Our findings demonstrate that few women (4.2%) used a condom during their first sexual intercourse. Another study showed that the prevalence of condom use at first sexual intercourse among Vietnamese adolescents and youths was 6.8% (17). This suggests that the first sexual intercourse among Vietnamese women carries a high risk of adverse SRH consequences. Considering the strong association between early sexual activity initiation and multiple sexual partners, low condom use is a further concern (17).

Our study results showed a weak relationship between educational attainment and multiple sexual partners. These results differed from the research conducted in other countries. A study in the United Kingdom found that young people who continued their education were more likely to have sexual intercourse later in life (18). In the United States, the proportion of 20–24-year-old women with less than high school education who had first sexual intercourse before age 20 was 95%, compared with 72% of those with some post-secondary education. The trend was similar in France, with 91% of women with the least education having the first sexual intercourse before age 20 compared with the 79–80% of women who completed high school (19). The findings of this study call for more focussed sexual health education in schools in Vietnam given that educational attainment alone does not appear to be sufficiently protective.

In this study, women living in urban areas had a greater likelihood of early sexual debut than those living in rural areas. Consistent with these results, a previous study in the United Kingdom also reported that the proportion of young women (under age 16), who had sexual intercourse was significantly higher among those with high levels of deprivation (based on housing type, education, employment) compared with women with low levels of deprivation (18).

Our finding that early sexual initiation was associated with women in Vietnam having multiple sexual partners over their lifetimes is comparable with the findings of research on this topic undertaken in Western countries. This is interesting, given that Vietnam has a reputation of being conservative in relation to sexual issues, although this is changing. De Sanjose et al. (20) reported that the first sexual debut before 16 years was associated with more than one sexual partner over the lifetime in Spain (OR=7.82 (95% CI:5.73–10.67)). A national survey in the United States conducted in 2002 had similar results, and it showed an inverse correlation between the number of partners and age at first sexual intercourse: 12–14 years, 14–15 years, 16–17 years, and 18–19 years with OR=7.5, 3.3, 2.3, and 1.4, respectively, with reference age of 20–24 years (21). One reason could be the short duration of relationships leading to the common exchange of sexual partners during adolescence and young adulthood (9). In the logistic regression analysis in our study, the age of the women was also a significant predictor of the number of sexual partners over their lifetimes, but this could be explained by noting that older women have more time to accumulate sexual partners. Although the findings of this study are similar to that reported in other populations, they provide useful insights into sexual behaviour among women living in a traditional society that, compared with many others, is less permissive regarding premarital sex or sex outside marriage.

## Strengths and limitations

Despite being the first study of its type, and having important policy implications, this study has some limitations. The analyses were conducted on cross-sectional data and therefore causality could not be established. In addition, it was not possible to analyse the trends over time, for example, in age at first sexual intercourse. Although we included a number of factors related both first to sexual initiation and multiple partners over lifetimes, there were a number of other potentially relevant factors, for example, related to women's families, for which we did not have data (22). Moreover, we investigated only one outcome, the binary variable, that is, multiple sexual partners. It is possible that women's health risks varied according to the number of sexual partners in given time periods. For example, two partners within 3 months may carry different risks that those from two partners within 3 years. However, we were unable to investigate this in the MICS data. Clearly, further research is needed to investigate risks more precisely and also sexual practices such as oral sex and homosexuality. Lastly, we acknowledge that there are other factors associated with the age at first sexual intercourse documented in literature that were not covered in this study. They include physiological and psychological factors, parent guidance and communication, and disposable income (18). These factors should be considered in further studies.

## Conclusions

The findings demonstrate that early sexual initiation among women in Vietnam was significantly associated with having multiple sexual partners over their lifetimes. Parents, teachers, and healthcare providers need to be aware that early sexual debut can be associated with subsequent unsafe sexual practices which can lead to greater health risks including HIV infection. This study provides evidence in support of educational interventions to increase safe sex practices among young women in Vietnam.
